# An abdominal-sacral approach with preoperative embolisation for vulvar solitary fibrous tumour: a case report

**DOI:** 10.1186/s12957-021-02206-5

**Published:** 2021-03-29

**Authors:** Akimasa Takahashi, Hiroki Nishimura, Tsukuru Amano, Mari Deguchi, Fumi Yoshino, Ryo Kasei, Fuminori Kimura, Suzuko Moritani, Takashi Murakami

**Affiliations:** 1 Department of Obstetrics and Gynecology, Shiga University of Medical Science, Setatsukinowa-cho, Otsu, Shiga 520-2192 Japan; 2 Department of Clinical Laboratory Medicine, Shiga University of Medical Science, Kusatsu, Japan

**Keywords:** Solitary fibrous tumour, Vulvar, Abdominal-sacral approach, Embolisation, Case report

## Abstract

**Background:**

Solitary fibrous tumours (SFTs) in the female genital tract are uncommon. Resection of these tumours is controversial because it can cause life-threatening haemorrhage. We report a case of vulvar SFT that was excised in a combined abdominal-sacral approach after preoperative embolisation.

**Case presentation:**

At another hospital, an inoperable intrapelvic tumour was diagnosed in a 34-year-old woman. Computed tomography and magnetic resonance imaging showed that the uterus, urinary bladder and rectum were compressed laterally by a pelvic tumour with a maximum diameter of 11 cm. This mass was hypervascular and had a well-defined border. Transperineal biopsy was performed, and immunostaining revealed that the mass was an SFT. The tumour was supplied by feeding vessels from the right iliac arteries. First, we embolised the feeding vessels. Second, we performed surgical resection in a combined abdominal-sacral approach; no blood transfusion was necessary, and no perioperative complications occurred. The final pathological diagnosis was SFT that was positive for CD34 and signal transducer and activator of transcription 6 according to immunohistochemical staining.

**Conclusion:**

During a year of follow-up, the disease did not recur. Treatment of pelvic SFT should aim at complete resection through various approaches after careful measures are taken to prevent haemorrhage.

## Background

Solitary fibrous tumours (SFTs) were first described by Klemperer and Rabin in 1931 as mesenchymal tumours of the pleura [1]. Although SFTs are commonly considered intrathoracic tumours, approximately 30% of them arise in various extrapleural sites [2, 3]. Of the extrapleural SFTs, those in the female genital tract are rare. This tumour is also characterised by low potential for malignant transformation and by abundant blood vessels. Surgical excision with curative intent is generally recommended for the management of this tumour, but controlling bleeding during the operation is often difficult [4, 5]. Surgical methods are controversial because tumour resection sometimes causes life-threatening haemorrhage.

We succeeded in complete en bloc resection of vulvar SFT, without morbidity or the need for blood transfusion, by a combined abdominal-sacral approach after embolisation of the vessels supplying blood to the tumour.

## Case presentation

**Fig. 1:**
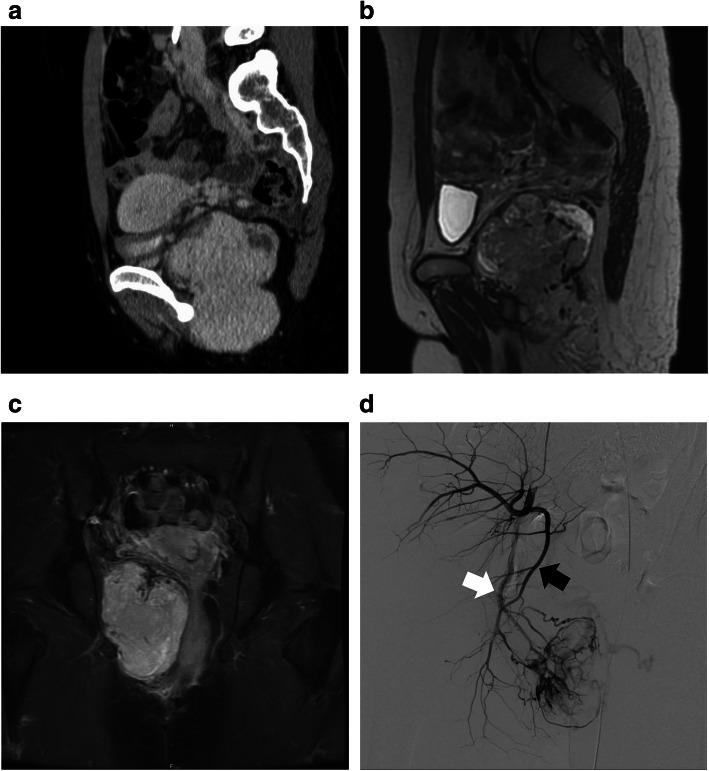
Preoperative imaging findings. **a** Preoperative contrast medium–enhanced sagittal computed tomography showed a pelvic mass lesion, measuring 112 × 62 × 58 mm. **b** T2-weighted sagittal magnetic resonance imaging (MRI) showed a mass with heterogeneous intensity in the pelvic cavity. **c** *Fat-suppressed* contrast medium–enhanced *T1*-*weighted c*oronal MRI showed a tumour with a relatively homogeneous contrast effect in the pelvic cavity. **d** On angiography of the right iliac artery, the solitary fibrous tumour was found to be supplied by the right obturator artery (black arrow) and the right internal pudendal artery (white arrow)

A 34-year-old woman was referred to our hospital to evaluate an asymptomatic pelvic mass detected with transvaginal ultrasonography in a private clinic, which she had visited for treatment of infertility. Computed tomography (CT) revealed a 112 × 62 × 58 mm hypervascular mass with a well-defined border. This mass compressed the bladder, uterus and rectum in the peritoneum (Fig. 1a). Subsequent contrast medium–enhanced and fat-suppressed T1-weighted magnetic resonance imaging (MRI) then revealed that the tumour had homogeneously high intensity, and T2-weighted images showed a mixture of isointensity in the muscles and high intensity of the tumour, as well as compression of the bladder, uterus and rectum (Fig. 1b, c). Contrast-enhanced CT and MRI revealed that the tumour was supplied with blood from the right pudendal artery. Laboratory data revealed no abnormalities such as squamous cell carcinoma antigen, carcinoembryonic antigen, cancer antigen 125, or carbohydrate antigen 19-9. We performed a transperineal biopsy, and the results of which established the diagnosis of SFT.

**Fig. 2:**
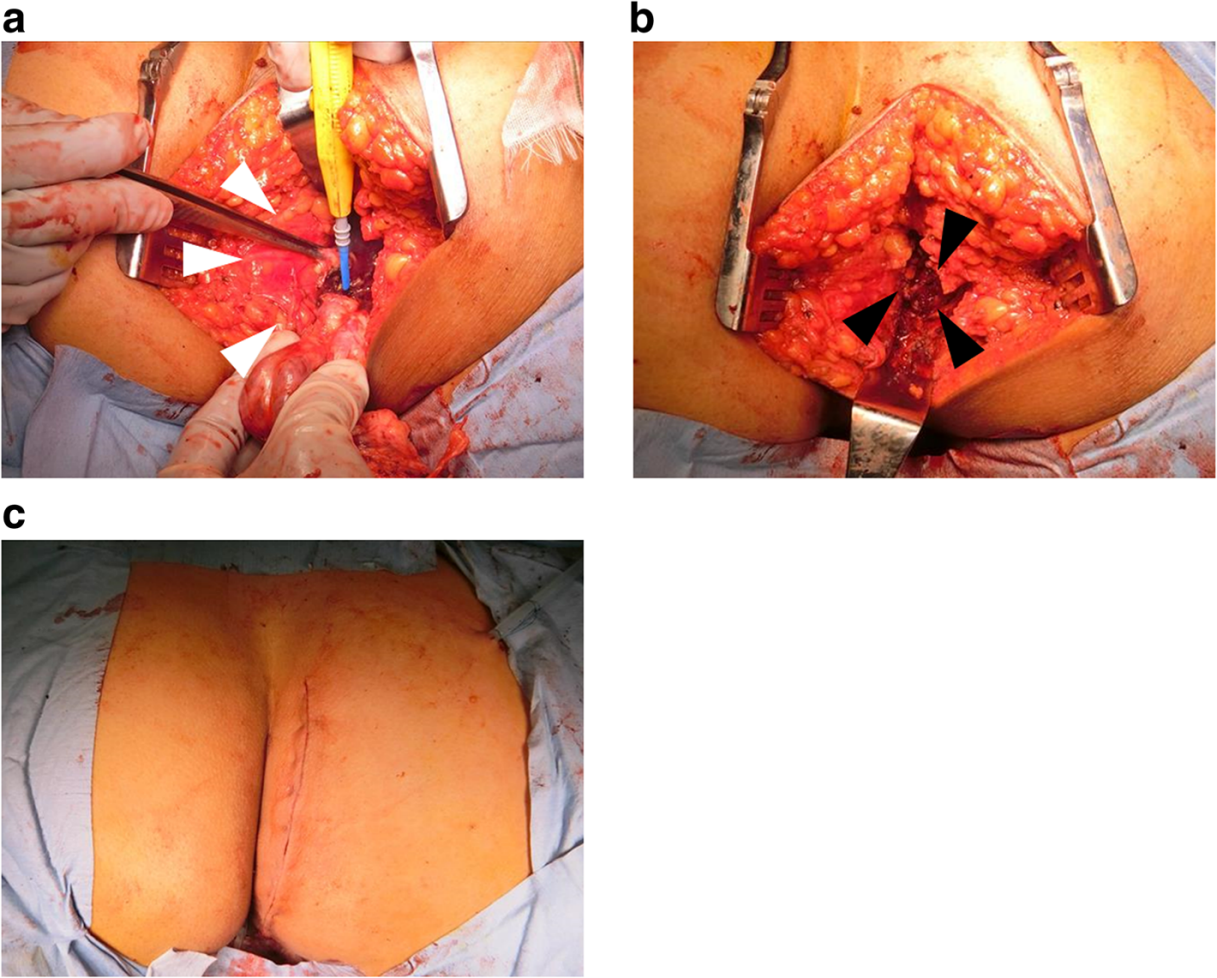
Intraoperative and postoperative findings of SFT from the sacral view, and postoperative wound. **a**, **b** En bloc resection of the tumour from the pelvic muscle fascia and rectum (white arrow: rectum, block arrow: abdominal cavity) **c** For tumour resection, the patient was in a jackknife position, and a lateral paramedian incision was made in the skin

Before surgery, we performed embolisation of the feeder vessels with an absorbent gelatin sponge to reduce intraoperative blood loss because this SFT was hypervascular, supplied primarily by the right obturator artery and the internal pudendal artery, according to angiography (Fig. 1d). The next day, with the patient in the supine position, we separated the tumour from the right side of the rectum and uterus through a transabdominal approach, which would have been challenging in a narrow and deep pelvis. Laparotomy was performed through a midline incision. We approached the paravesical space and confirmed that the tumour invaded the retroperitoneal cavity. The tumour was well encapsulated and had abundant blood vessels around the tumour. We separated as much of the tumour as possible from the levator ani. It could be easily separated from the surrounding muscles. After the wound was closed, the patient was repositioned into the jackknife position for resection through the sacral approach. We made a paramedian skin incision and easily identified the elastic but hard tumour (Fig. 2). We ensured the adequacy of surgical margins to prevent local recurrence and minimise bleeding. That is, the adipose tissue is left to cover the lesion. Finally, the tumour could be removed with en bloc resection. The tumour was completely excised over a period of 223 min with 250-mL blood loss, and no blood transfusion was required. We did not place the drain anywhere. The postoperative course was uneventful, and she was discharged on postoperative day 7.

**Fig. 3:**
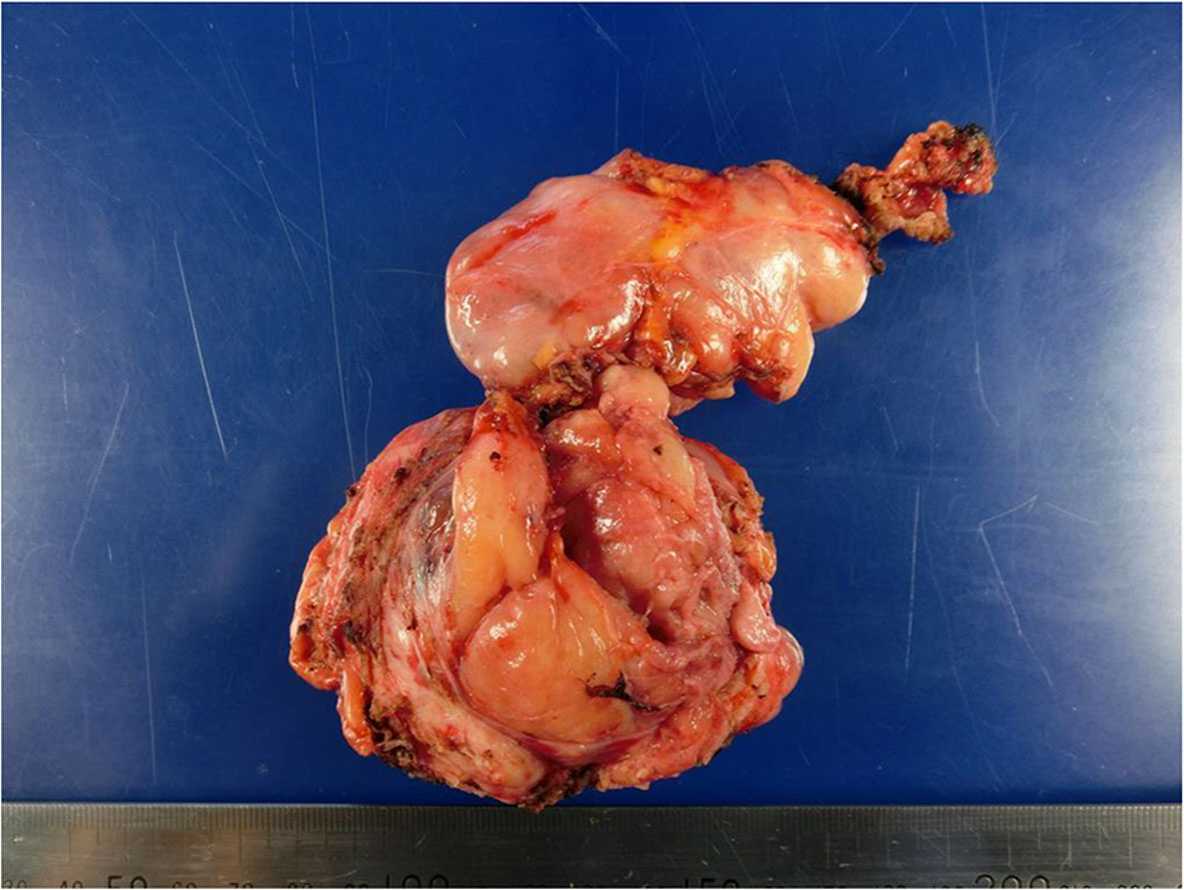
Gross of solitary fibrous tumour. Photograph of the tumour. Macroscopically, the tumour was elastic but hard, with an intact capsule and the cut surface was greyish-white

**Fig. 4:**
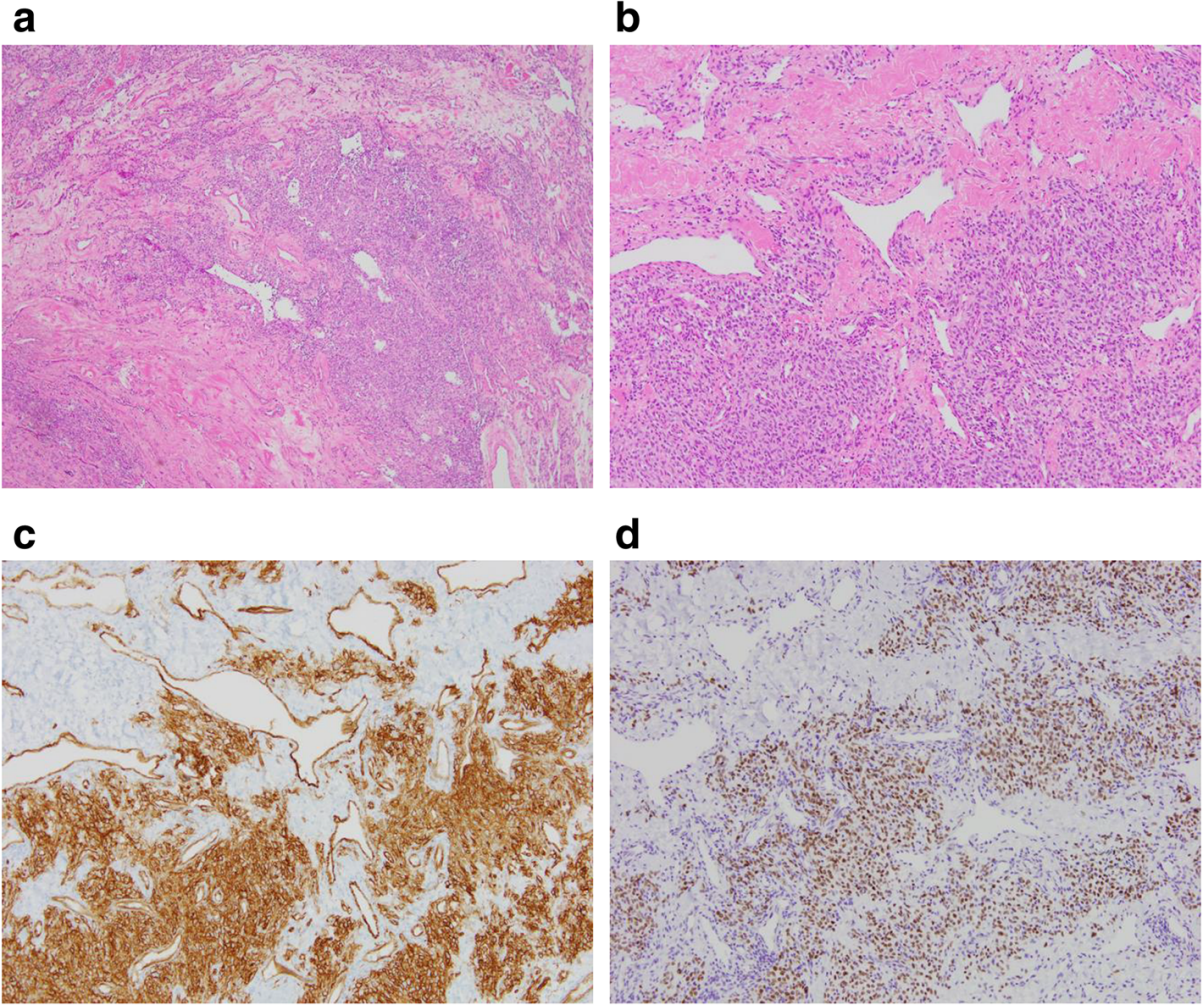
Microscopic histological findings. **a**, **b** Microscopic findings showed spindle cells with a patternless growth arrangement and enlarged blood vessels. (**a** ×40 magnification, **b** ×100 magnification; haematoxylin and eosin stain). **c** The tumour cells were strongly positive for CD34 (×100 magnification). **d** The tumour cells were positive for signal transducer and activator of transcription 6. (×100 magnification)

Postoperative pathological study showed that the tumour was encapsulated and elastic but hard, and the cut surface was greyish-white (Fig. 3). Microscopic study confirmed that the tumour capsule was not ruptured and was covered with adipose tissue. It was then revealed that the tumour consisted of proliferating, relatively small oval and spindle cells with prominent branching and a hemangiopericytoma-like vascular pattern. Cytological atypia was not significant. Immunohistochemical staining revealed that the tumour cells were positive for CD34 and for signal transducer and activator of transcription 6 (STAT6) (Fig. 4). The final diagnosis was also SFT. The postoperative course was uneventful; no adjuvant treatment was given because complete surgical resection was achieved. In the year since surgery, the patient has shown no evidence of tumour recurrence.

## Discussion and conclusions

In this case, a solitary fibrous tumour invading the retroperitoneum from the vulva was treated successfully by a combined abdominal-sacral approach after embolisation, without a need of blood transfusion. To our knowledge, this is the first case report of the resection of a vulvar SFT through a combined abdominal-sacral approach.

SFTs are rare soft tissue tumours that commonly arise in the pleura [1]. Such tumours rarely arise from the female genital tract, although they have been reported in various other organs. Nine percent of SFTs occur in the female genital tract, and only 42 cases, including several in the retroperitoneum, have been reported so far [5, 6]. Furthermore, only 11 cases of vulvar SFTs have been reported [7]. The management of vulvar SFTs is controversial: The prognosis depends on complete resection of both extrapleural and pleural SFTs [5], but surgery is difficult because of frequent intraoperative heavy bleeding, which occurs because SFTs in the pelvis are usually supplied with blood by multiple vessels, such as the branches of the inferior mesenteric artery or the internal iliac arteries [8, 9]. Therefore, it is necessary not only to ensure a sufficient blood supply but also to control bleeding during surgery.

SFTs are soft tissue tumours with spindle-shaped cells of mesenchymal origin. From among CD34 immunostaining positive soft tissue tumours, we need to differentiate between SFT and dedifferentiated liposarcoma. Immunohistochemistry for STAT6 protein is useful for diagnosing SFT [10]. Strong expression of STAT6 in the nucleus is an ideal diagnostic indicator for SFT with high sensitivity and specificity [11]. It has been recently reported that STAT6 stains heterogeneously in the nucleus and cytoplasm in spindle-cell de novo dedifferentiated SFT, and the nerve growth factor-inducible protein A binding protein 2 (NAB2)-STAT6 gene fusion was confirmed in this tumour. In our case, the tumour showed the classical morphology of spindle-shaped cells with CD34 staining [12]. Moreover, STAT6 was uniformly and strongly expressed in the nucleus of these cells. Therefore, we diagnosed this tumour as a SFT.

Because the tumour extended from the vulva into the pelvis, we performed the surgery through a combined abdominal-sacral approach out of concern about the difficulty in establishing an appropriate surgical field deep inside the pelvis by laparotomy. For that reason, we first separated the tumour from the right side of the rectum and uterus through a transabdominal approach and then successfully resected the tumour through a transsacral approach. Most patients with pelvic SFTs have undergone laparotomy, but some patients suffer heavy bleeding, which is difficult to control [9, 13]. In one report, massive bleeding was not avoided even with the transperineal approach [14]. Katsuno et al. reported that the transsacral approach was useful for complete resection of pelvic SFTs [15]; however, this approach carries a high risk of postoperative complications, such as surgical site infection and *anal dysfunction* [16] (Table 1). In another report, surgery with a combined abdominal-sacral approach was performed for five cases of giant presacral tumours, and complete resection without massive bleeding was achieved. The advantages of this approach are that complications are minimised and it allows for complete resection of a tumour that may be difficult to remove through other approaches [19]. Thus, a combined abdominal-sacral approach can be an option for resecting tumours deep in the pelvis.

**Table 1:** Summary of surgical outcomes of solitary fibrous tumours in the female pelvis

Author	Age	Tumor size (cm)	Way of operation	Estimated blood loss (g)	Complication	Follow-up (months)
Wat, et al. [13]	63	14x11x14	Laparotomy	8,000	Blood transfusion	N/A
Soda, et al. [9]	27	16x9x14	Laparotomy	13,660	Blood transfusion, aortic balloon catheter	Free of disease 1 year after the excision
Katsuno, et al. [15]	56	9x7.5x5	Trans-sacral approach	267	No	Free of disease 20 months after the excision
Kim, et al. [4]	52	12x9x9	Laparotomy	Massive hemorrhage	Rebleeding→reoperation	Free of disease 3 years after the excision
Fard-Aghaie, et al. [17]	70	19x14x9	TAE→Abdominoperineal approach	Less than 200	Permanent colostomy	Free of disease 13 months after the excision
Yuza, et al. [18]	46	17	TAE→Laparotomy	335	Ileostomy	Free of disease 2 years after the excision
Present case	34	11x6x6	TAE→Abdominal-sacral approach	250	No	Free of disease 6 months after the excision

Abbreviation: *TAE* trans-arterial embolisation

We embolised feeder vessels to the tumour before surgery to reduce intraoperative bleeding. Preoperative percutaneous arterial embolisation allows for safe and complete resection in cervical, thoracic and lumbar locations in the spinal cord [20, 21]. Embolisation for pelvic SFT has been reported; Soda et al. reported that a tumour was resected after blood flow block was achieved by an intraoperatively inserted aortic balloon catheter, and the resulting blood loss was 13,660 mL [9]. On the other hand, in other reports, the feeder vessels of SFTs were selectively embolised before operation, which resulted in less intraoperative blood loss without the need for blood transfusion [14, 17, 22, 23]. In addition, in two reports, preoperative embolisation did not have the effect of shrinking the tumour [17, 22]. In our case, we used an absorbable gelatin sponge that has been reported to be pregnant after using it for uterine artery embolisation because she desired for a baby. It is known to be absorbed within 2–6 weeks [18]; therefore, it is reasonable to perform surgery within 2 weeks. Therefore, we performed surgery the day after embolisation. It was possible to complete surgery without blood transfusion by performing preoperative embolisation. Therefore, embolisation may control intraoperative bleeding, but it is not effective in reducing tumour volume. In addition, selective embolisation of the feeding vessels is more appropriate than intraoperative aortic occlusion.

In summary, we completely resected a vulvar SFT without blood transfusion. This tumour is very rare, nonmetastatic and characterised by abundant blood vessels. The main treatment for SFTs is surgical resection. However, pelvic SFTs carries the risk of massive bleeding and organ damage, and inadequate tumour resection can lead to local recurrence. Preoperative embolisation of feeder arteries reduced intraoperative bleeding in our patient. In addition, use of the abdominal-sacral approach can reduce perioperative complications. This combination thus has potential in the treatment of pelvic SFTs.

## Abbreviations

CT: Computed tomography
MRI: Magnetic resonance imaging
SFT: Solitary fibrous tumours
CD: Cluste of differentiation
STAT6: Signal transducer and activator of transcription 6
